# Strong Noise Rejection in VLC Links under Realistic Conditions through a Real-Time SDR Front-End

**DOI:** 10.3390/s23031594

**Published:** 2023-02-01

**Authors:** Muhammad Ali Umair, Marco Meucci, Jacopo Catani

**Affiliations:** 1 European Laboratory for Non-Linear Spectroscopy (LENS), University of Florence, I-50019 Sesto Fiorentino, Italy; 2 National Institute of Optics-CNR (CNR-INO), I-50019 Sesto Fiorentino, Italy

**Keywords:** visible light communication (VLC), wireless communication, intelligent transportation systems, Li-Fi, software-defined radios

## Abstract

One of the main challenges in the deployment of visible light communication (VLC) in realistic application fields, such as intelligent transportation systems (ITSs), is represented by the presence of large background noise levels on top of the optical signal carrying the digital information. A versatile and effective digital filtering technique is, hence, crucial to face such an issue in an effective way. In this paper, we present an extensive experimental evaluation of a complete VLC system, embedding a software-defined-radio (SDR)-based digital signal processing (DSP) filter stage, which is tested either indoors, in the presence of strong artificial 100-Hz stray illumination, and outdoors, under direct sunlight. The system employs low-power automotive LED lamps, and it is tested for baud rates up to 1 Mbaud. We experimentally demonstrate that the use of the DSP technique improves 10× the performance of the VLC receiver over the original system without the filtering stage, reporting a very effective rejection of both 100-Hz and solar noise background. Indoors, the noise margin in the presence of strong 100-Hz noise is increased by up to 40 dB, whilst in the outdoor configuration, the system is capable of maintaining error-free communication in direct sunlight conditions, up to 7.5 m, improving the distance by a factor of 1.6 compared to the case without filtering. We believe that the proposed system is a very effective solution for the suppression of various types of noise effects in a large set of VLC applications.

## 1. Introduction

In the era of 5G (and beyond) networks, VLC has already proved itself as a suitable candidate for future wireless communications, toward the implementation of smart cities and IoT protocols requiring either high data rates, pervasive connections, low latency, and low energy consumption [1,2]. By employing ubiquitous LED sources, VLC could enable common illumination and signaling infrastructures for simultaneous illumination and communication, with reduced costs and energy consumption [3]. VLC applications widely span from outdoor to indoor scenarios, as vehicle-to-vehicle communication (V2V) [4], light-fidelity (Li-Fi) [5], indoor VLC links [6], and VLC positioning systems [7]. Recent works have demonstrated VLC as a reliable technology for short-range [8] and mid- to long-range communications [9,10]; integration with the 5G network has recently been reported [2].

However, background noise induced by stray artificial illumination and/or strong solar irradiance could be particularly detrimental in realistic VLC implementations, strongly limiting the quality and haul of the communication link. For these reasons, it is highly desirable to provide a VLC receiver stage with dedicated filter units to reduce the effects of environmental noise and to recover a sufficient signal-to-noise (SNR) value. A very common approach is to employ a bandpass optical filter mounted in front of a photodiode (PD), allowing only certain wavelengths to be collected by the PD [11,12]. Such a technique is also employed to increase the VLC bandwidth when white LEDs are used [13], by rejecting the slow yellow-converted spectral component of the LED, typically featuring a few-MHz bandwidths [14]. However, this approach does not exploit the total optical power of the LED source [15], which could instead be essential to achieve long hauls when ultra-large bandwidths are not necessary. Consequently, the employment of a suitable electronic filtering stage is very desired in real-life VLC applications, especially when white LED light sources are employed, as optical filtering would not bring practical benefit.

Solutions relying on electronic filtering of stray components are reported in [16], where authors have implemented analog filter schemes to filter out undesired voltage levels at the receiver side after the trans-impedance (TIA) stage. The simulated front-end includes automatic gain control (AGC) to maintain output signal around acceptable values. In other works [17] a static, matched filtering stage is applied between PD and TIA to reject the low-frequency noise due to sunlight and attenuates other artificial illumination sources for up to 30 dB. More advanced filtering schemes relying on active and/or digital front-ends have recently been reported either using matched filter [18] schemes or bandpass filtering [19]. Papers [20,21] report adaptive filtering stages based on automatic gain control (AGC) blocks. The authors proposed an open-loop signal processing technique in which SNR is analyzed in real time, based on the SNR analysis, the receiver updates its structural design to keep optimum performance. However, the performance of the receiver is evaluated only through simulations, and there are no implementations in realistic systems. In [11], the authors proposed and simulated an analytical model for MISO based VLC system, showing that the performance of the system improves significantly in the presence of environmentally induced optical interference, achieving a forward error correction threshold.

In recent years, software-defined radios (SDRs) have gained a lot of attention from academia and industry [22,23], mainly due to the versatile combination of hardware and software modules making such systems excellent tools for designing and testing complex communication systems. Benefiting from the RF community, which has extensively used the concept of SDR [24,25], such technology has recently been exploited in VLC applications, in indoor, outdoor, and vehicular applications [26,27]. However, these works have shown the performances of the VLC system without a comparative evaluation of the improvement that a SDR front-end could deliver over existing VLC systems, which is especially relevant in realistic scenarios such as in the presence of background noise and/or strong solar irradiance.

In this paper, we present a complete VLC system embedding a new real-time digital signal processing (DSP) filter stage realized using SDR. The performance of the system, as well as the benefits of the new SDR-based stage, are experimentally assessed in realistic conditions with commercial, low-power LED sources, in indoor scenarios (in the presence of a strong 100 Hz background light), and outdoor scenarios under direct sunlight exposure. We compare the performances of the SDR-based system with those of a standard digital system without SDR-based DSP stages, finding that our SDR-based DSP stage improves by up to 40 dB of the SNR noise margin in the presence of large background artificial illumination for indoor applications. Moreover, we perform outdoor tests, showing error-free links under direct sunlight exposure for distances up to 7.5 m, which is 1.6 larger than the link length obtained without the SDR-based DSP stage.

The paper is organized as follows. Section 2 describes the details of the SDR-based VLC system, as well as the experimental campaign performed. Section 3 reports on the methods used for evaluating the performance of our system. Section 4 presents a thorough description of the experimental campaign in both indoor and outdoor scenarios, along with a discussion of the results, and Section 5 presents our conclusions and outlook for our work.

## 2. Hardware and Methods Overview

In this section, we first provide an overview of the hardware and experimental setup (Section 2.1), then a description of the configurations used follows (Section 2.2) and, finally, the experimental campaign carried out in indoor and outdoor scenarios is presented (Section 2.3).

### 2.1. Hardware and Experimental Setup

**Figure 1:**
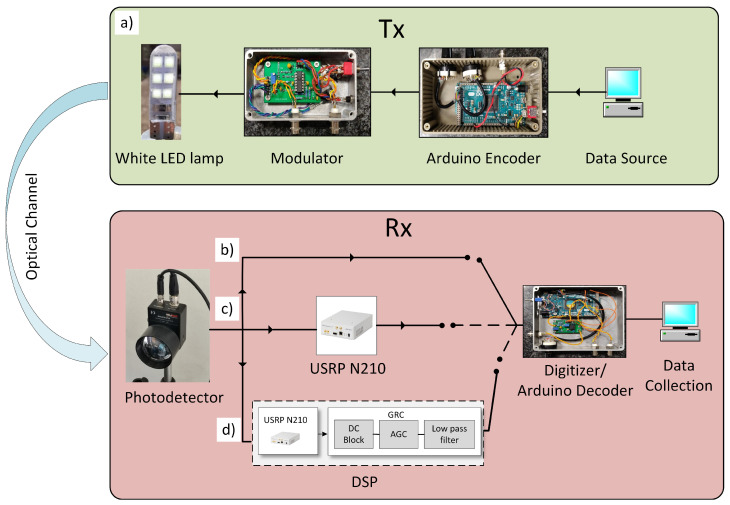
VLC system block diagram: (**a**) The upper panel shows the block diagram of TX hardware, including the data packet generator and current modulator, which supplies the automotive LED lamp. The lower panel shows the VLC RX hardware system used for the three configurations analyzed (see text): (**b**) *Direct Arduino System*, (**c**) *Bridge System*, and (**d**) *Full System*. For all three configurations, the digitization and decoding process is performed by the Arduino RX Digitizer/Decoder board, which also performs a byte-wise comparison for PER calculation.

The core of the setup in which the SDR DSP stage is integrated is based on a low-cost, open-source Arduino DUE platform (for full details see [28], see Figure 1 for a block diagram). The VLC transmitter board (TX) constructs a continuous stream of $10^{5}$ packets using on–off keying (OOK) modulation scheme at 1 Mbaud with Manchester encoding.

It inputs the modulated data stream to a custom-made current modulator with 2 MHz bandwidth which is used to provide the DC current to turn on the white LED (160 mA) and the AC current profile to provide intensity modulation (0–200%) on the white LED source intensity. The low-power LED used as the VLC light source (V5W, 12 V, 6000 K color temperature, shown in Figure 1 on TX block) is widely employed in automotive sector. In our experiments, two different photodetectors are used as receiving stages (RXs): Thorlabs PDA100A2 and APD430A/M, respectively. An aspheric condenser lens (ACL25416U, focal length f = 16 mm) is mounted in front of the PD to increase the optical gain and reduce the field of view (FoV) of the RX stage (see Figure 1, RX block). The light source and detector were fixed at the same height as that of the white LED (about 1.1 m from the floor) maintaining a direct Line of Sight (LoS) between TX and RX.

The PD detects the optical signal carrying the digital VLC information and converts it into voltage.

In the original communication system based on Arduino (Figure 1b), the signal is fed to the input of the Arduino-based receiver (RX) where it is first converted into digital form by a Schmitt-trigger physical comparator with few-mV hysteresis to reduce false-triggering events induced by the noise. The digitized signal is then analyzed in real-time by the Arduino DUE RX system, which detects the PER by counting the number of correctly received packets over the total amount of $10^{5}$–$10^{6}$ sent packets, depending on the experimental run. We, hence, take PER $\leq 10^{-5}$ as the error-free threshold. A packet is considered lost when one or more bits are recorded as altered with respect to the bit stream composing a reference message, and in the low-PER regime, this corresponds to a bit lost per packet lost. Since our packets are composed of 32 bits, the error-free threshold for BER is $3.1\times 10^{-7}$.

### 2.2. RX Stage Configurations

Whilst the configuration of TX is kept unchanged, we implemented and tested three different configurations of the RX stage (see Figure 1). The *Direct Arduino System* corresponds the original configuration discussed in [28], where the PD and the input of the Arduino-based RX stage are connected directly so that the digitization of the signal is performed by the RX board itself (see Figure 1a). Figure 1b shows the *bridge system*, in which the SDR USRP N210 is inserted between the PD output and the RX block input. SDR is equipped with software that simply replicates the input signal to the output. In this case, SDR is used to replicate the PD signal and to bridge it toward the comparator stage of the RX board. This configuration is useful to quantify the excess noise which is injected into the RX stage by the presence of an additional SDR stage. Finally, Figure 1c shows our newly designed *full system* in which a full-fledged SDR-based filter stage is active between PD and discriminator at RX. By means of the SDR-based system, we can implement complex filtering and DSP blocks running on a FPGA-based SDR board. The hardware part of SDR exploits an Ettus Research USRP-N210 [29] system with 30 MHz and RFTx daughter boards [30]. The SDR-based digital signal processing (DSP) stage [31] is implemented using the open-source GNU radio companion (GRC) software [32]. The sampling rate is set to 20 Ms/s, limited by the data handling capabilities of the USRP boards. In GRC, the first DC-block stage (Figure 1d) performs the moving average of the incoming signal, rejecting the low-frequency components of a −3 dB high-pass frequency of 10–100 kHz, depending on the used baud rate realization. After the DC block, an AGC block with a 1 ms update rate and maximum gain = 10,000 is used to dynamically amplify the received signal before it is fed into a low-pass filter block with a cut-off frequency of 4 MHz. The signal is then digitized by a software discriminator block with an adjustable threshold, providing a TTL logic signal which is then passed to the Arduino RX board.

### 2.3. Experimental Procedure and Scenarios

**Figure 2:**
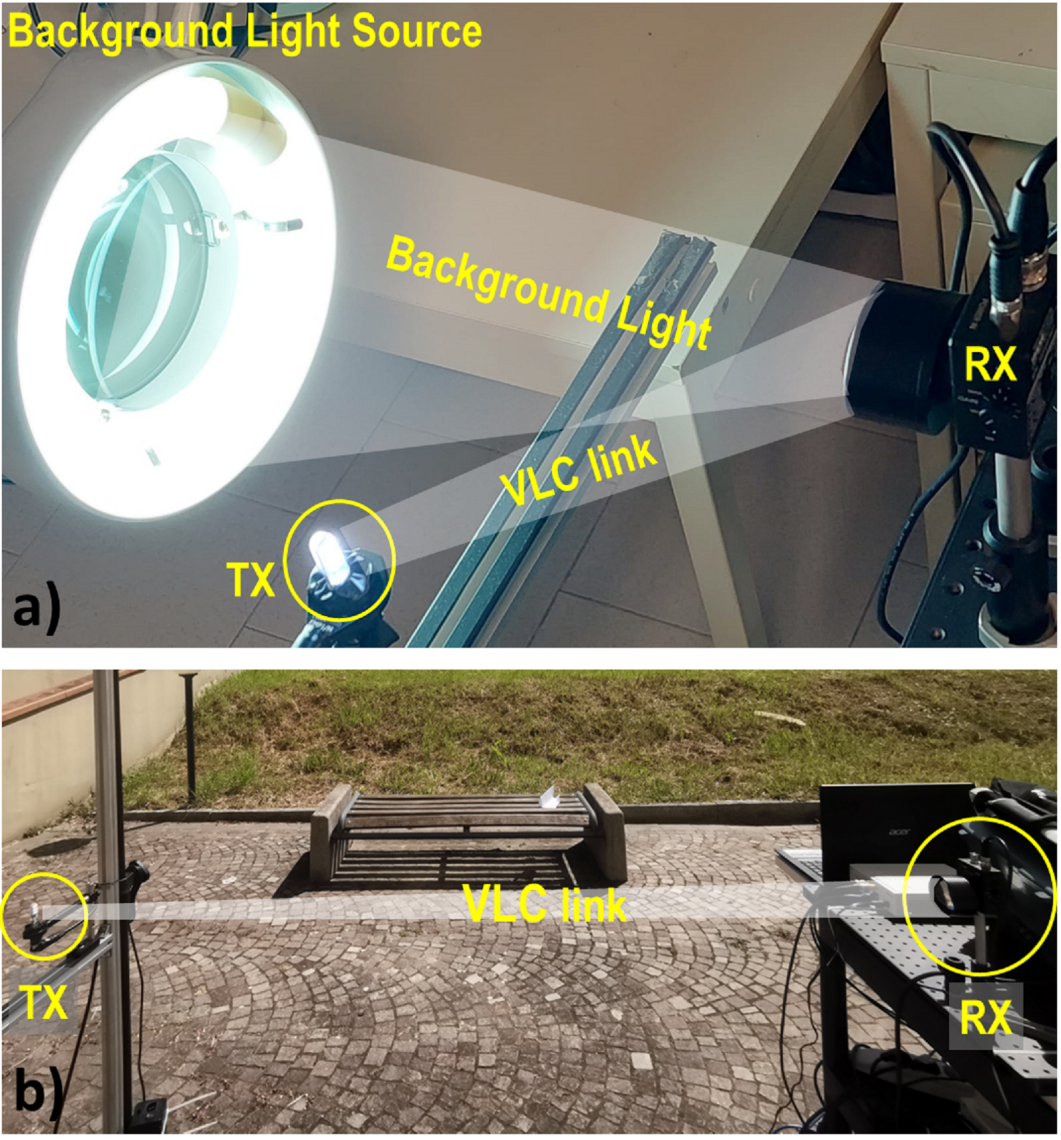
Experimental setups used: (**a**) Indoor measurements in the presence of strong background light, (**b**) outdoor measurements setup under strong solar irradiance.

In our experiments, the amplitude of the received signal and the relative SNR level can be measured at various points of the chain, either via dedicated software blocks in the SDR system (see Section 4), either by a 4-channel Tektronix digital oscilloscope (MDO3024). The optical signal (OS) amplified by PD is fed to the Universal Software Radio Peripheral (USRP) to be processed in real time. As a first experimental test, we compared the three setups in order to highlight the improvements introduced and refine the filtering software. The results of this first analysis are reported in Section 4.1. Subsequently, we analyze two scenarios that often can occur in the implementation of VLC links. In Section 4.2, an indoor scenario is analyzed. In this scenario, we analyze the effect on the VLC communication link exerted by the presence of strong 100-Hz stray lights. An image of the indoor experimental setup is shown in Figure 2a. In Section 4.3 we instead demonstrate the potential of the system in an Outdoor environment, under the presence of strong solar irradiance. The outdoor experiment was carried out around 12 am–2 pm when the solar irradiance was at its peak. An image of the outdoor experimental setup is shown in Figure 2b.

## 3. Model and Data Analysis

In this section, we give details regarding the data analysis and the mathematical expression used for evaluating the performances of our system. Assuming a uniform distribution of the error bits on bad packets, PER can be related to the bit error rate (BER) via $\mathrm{PER}=1-{\left(1-\mathrm{BER}\right)}^{N}$ [33], where *N* represents the number of bits in a single packet that in our case is N=32. Considering the well-known relation between BER and SNR, valid in the case of OOK modulation and additive white Gaussian noise (AWGN) channel, we can integrate in the equation $\mathrm{PER}=1-{\left[1-0.5\;erfc\left(E_{b}/N_{o}\right)\right]}^{N}$, where $E_{o}$ is energy carried by a single bit and $N_{o}$ is noise power spectral density.

The above expression can be written as:

(1) $$\mathrm{PER}=1-{\left[1-0.5\;erfc\left(\frac{a(x-x_{c})}{2\sqrt{r}w}\right)\right]}^{N}$$

In (1), *x* and $x_{c}$ are the amplitude of the measured signal and the threshold value, respectively, at the comparator stage. α is a rescaling factor taking into consideration the different gain values of USRP and input impedance values of the three setups, making identical OSs emerge with different values after the PD stage. *w* is the RMS value of noise at the discriminator (either hardware or software depending on the configuration), and *r* is a factor of 1 for a square signal (as in our case) and 2 for a sinusoidal (bandwidth-limited) one.

## 4. Experimental Campaign and Discussion of Results

Firstly, in Section 4.1, the system was tested indoors with artificial lights turned off to compare the performances of all three systems. Subsequently, in Section 4.2, the effectiveness of our SDR-base DSP stage in the rejection of artificial light is demonstrated. Finally, in Section 4.3, outdoor communication in the presence of high solar irradiance is demonstrated and characterized.

### 4.1. Comparison of the Three Systems: Indoor Tests

**Figure 3:**
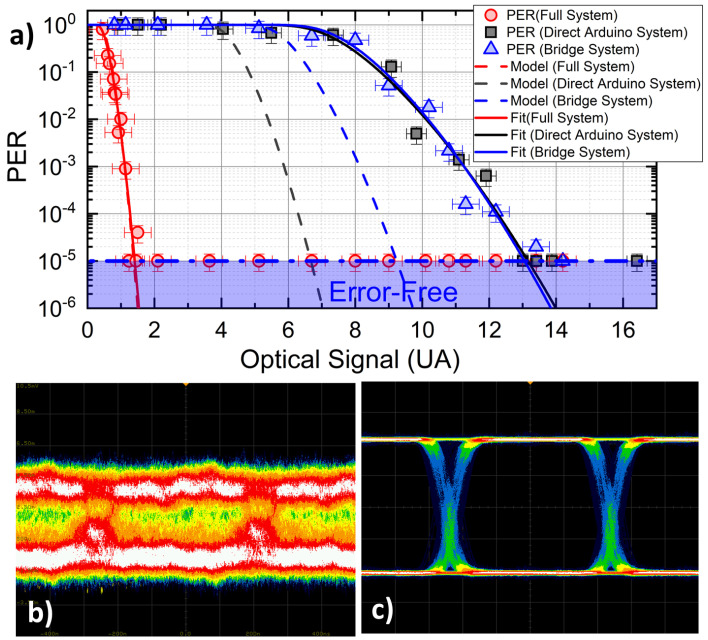
(**a**) PER as a function of the optical signal (OS): comparison between the three experimental systems used. (**b**) Eye diagrams related to OS = 5 AU case of the panel recorded for direct PD output and (**c**) after SDR output (*full system*).

Indoor tests are used to quantify the maximum performance limits of the VLC communication setup, and the capability of the SDR-based filter stage to provide for improved rejection of artificial light interference on the VLC link electronic noise.To this scope, we compare how the three systems behave in terms of PER for a given OS amplitude. Figure 3a shows the PER for all of the three configurations (filled symbols), recorded as a function of OS, which has been varied by controlling the amount of LED light collected by the PD. In order to compare the performances of the systems in equivalent conditions, we normalize the signal amplitude recorded by the PD stage in the three configurations after compensation of the different impedance and electronic amplification factors which could differ case by case (factor α in Table 1). The comparison highlights a very strong enhancement of the sensitivity of the RX stage introduced by the SDR stage, which allows for error-free communications for OS values (red symbols) as low as 1/10 of the original value (black symbols). This improvement is made evident also by the eye patterns reported in Figure 3b,c. The *bridge* configuration does not introduce significant discrepancies with respect to the original system, as expected, meaning that the excess noise introduced by the SDR block is negligible as compared to the optical channel noise affecting the VLC link. The dashed lines in Figure 3a report the predictions of the model discussed in Section 3. Experimental parameters used for modeling are reported in Table 1. In both the *Direct Arduino System* and *bridge system* case, noise RMS values *w* appearing in Equation (1) are directly measured before the comparator stage of the RX board via the digital oscilloscope, whereas in the *full system*, noise RMS is measured before the (software) discriminator block, with a dedicated software block.

Whilst the predictions of the model are in excellent agreement with data for the *Full* configuration, the model features a sizeable discrepancy in the *Bridge* and *Direct Arduino* configurations. This behavior stems from an additional form of uncertainty, affecting our Arduino-based RX discriminator, whose contribution is not considered in our model. In both *Direct Arduino* and *Bridge* systems, the discrimination is performed by the physical comparator stage. Since the signal level could attain small values in such configurations, residual self-oscillation phenomena can lead to false triggering and jitters in the digitized signal, which is fed to the Arduino. This noise excess is suppressed in the *full* system, where the software discriminator acts after several filtering stages, and always delivers a large logic signal of 0–1 V as input to the physical comparator.

Our data are also plotted against the predictions of a fit, computed by leaving either *w* and/or $x_{c}$ as free parameters in Equation (1). The best-fitting parameters are given in Table 1 for all three cases. As anticipated earlier, the configuration where the SDR DSP stage is *not* active basically acts as the original system where an extra noise factor (in the range of 1.5–2.5 times the original value of *w*) is added. Interestingly enough, instead, the SDR case is perfectly reproduced by a model without the need for free parameters.

### 4.2. Rejection of Artificial Light Contribution

**Figure 4:**
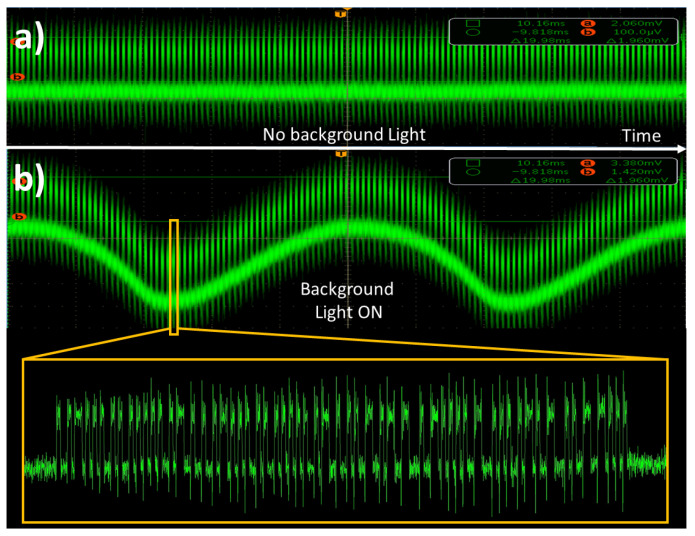
Example of OS after PD amplification with artificial lights off (**a**) and on (**b**), respectively.

In the deployment of indoor VLC technology, a critical issue is represented by the presence of low-frequency interference caused by common fluorescent lights, typically producing 100 Hz (and harmonics) periodic noise in the received signal, and by slow movements of people and objects in the room. Moreover, fluorescent lamp drivers often feature an AC-switching frequency of 100–500 kHz which could severely hamper the quality of VLC links if filter stages are not properly designed. This effect could be particularly detrimental for low received signal amplitudes, where noise-induced fluctuations could bring the signal out of the digitization threshold of the physical comparator (see Figure 4).

**Figure 5:**
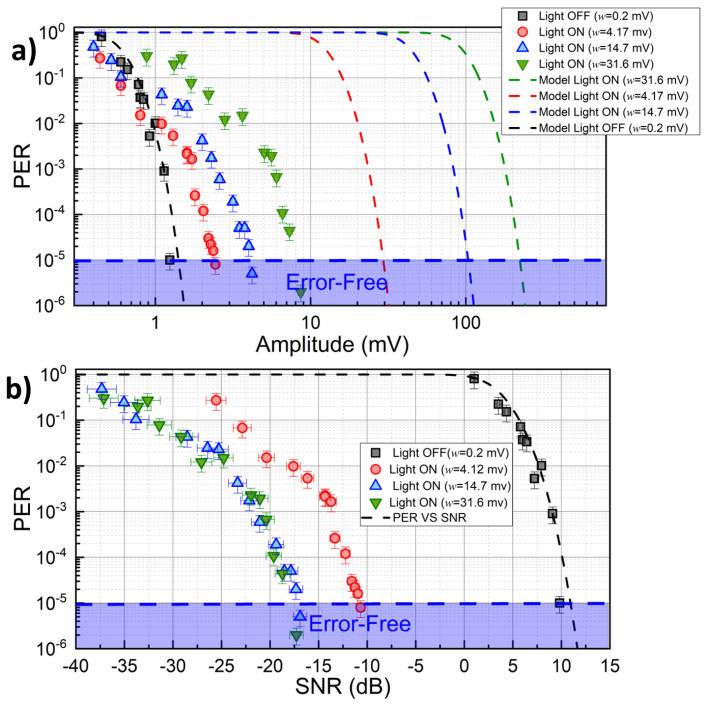
Effect of indoor background light intensity on communication quality in terms of PER, for 3 different levels of background illuminance: (**a**) PER vs. signal amplitude at the receiver, (**b**) PER vs. measured SNR.

We record the PER for our *full* system in the presence of a variable amount of background light intensity emitted by a neon lamp and collected by the detector (see Figure 2a). Results of such analysis are reported in Figure 5a, for three different noise levels *w* (4.17 mV, 14.7 mV, and 31.6 mV, respectively) set by adjusting the distance between the neon light and PD. Black symbols correspond to background lights off. The dashed lines show the predictions of Equation (1) evaluated for each case with the corresponding value of *w* measured at the PD output. These lines correspond to the ideal behavior of the VLC system in different noise conditions in case the signal would not be filtered by SDR blocks. As can be seen from Figure, the *Full* configuration provides error-free communications also in the presence of background RMS noise larger than three times the optical signal amplitude (*w* = 31.6 mV). The beneficial effect of the DSP stage is even clearer in Figure 5b, where PER is reported as a function of the SNR after the PD stage. In the worst noise scenario (green symbols), a noise margin of −17 dB is observed in the low-PER region (PER $=1\times 10^{-5}$). For comparison, black dots/dashed line report the data/model predictions for the case of no background lights, where the required noise margin is +10 dB. Our data show that the improvement in the noise margin for large noise always exceeds 27 dB in the low-PER case, and improves further to reach 40 dB in the large-PER region (PER $=1\times 10^{-1}$).

### 4.3. Outdoor Tests

We carried out the outdoor analysis of the VLC link quality testing the system under direct solar irradiance (see Figure 2b), for typical luminous fluxes of 1.2 × 10${}^{4}$ lux, representing a very demanding challenge for real-life VLC applications. In our characterization, we compare the performances of *Direct Arduino* and *full* systems. As shown in Section 4.1, the *Bridge* configuration has not shown significant discrepancies with respect to the original system, so it was not considered in this analysis. The APD Thorlabs APD430A/M due to its sensitivity was used in this measurement set. The presence of large background solar irradiance introduces a large shot-noise contribution in the photocurrent [34], with a white spectrum whose filtering-out process is, in general, more demanding as compared to the indoor case where the largest contribution of noise is concentrated in the low-frequency region below 1 kHz.

**Figure 6:**
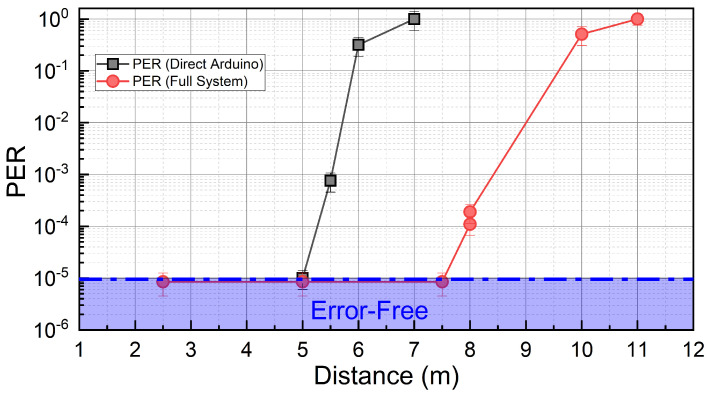
Outdoor tests: PER as a function of the distance between TX and RX, under a solar irradiance of 12,000 lux. Results were obtained with the traditional system (black) and the *full* sDR system (red).

Figure 6 shows the comparison PER as a function of the distance between the TX and RX stages, for the two systems. The presence of a low-frequency cutoff at the PD stage [35] allows also the *Direct Arduino* system to achieve error-free communication distances up to 5 m. However, with the *full* system, the error-free distance (7.5 m) is increased by 1.6 times, and successful communications are observed up to 10 m. This is a remarkable result, as the low-power V5W employed LED is very common in the automotive industry, demonstrating that realistic VLC ITS protocols could be virtually retro-implemented in existing cars without the need for dedicated high-power LED sources. We also remark that, by decreasing the baud rate, larger $E_{b}/N_{o}$ ratios could be achieved at a given distance (see Section 3), and larger error-free distances are expected.

## 5. Conclusions

In this work, we reported an extensive indoor and outdoor characterization of a new VLC system embedding a digital DSP filter stage built on SDR technology, for the active and dynamical removal of environmental background noise. Specifically, we couple the DSP filter stage with a low-cost VLC prototype based on Arduino previously developed, driving a commercial, low-power V5W white automotive LED, to experimentally demonstrate and comparatively quantify, for the first time, the improvement brought by an SDR-based DSP stage in realistic indoor and outdoor (direct sunlight) VLC applications, for a baud rate of 1 Mbaud. We provide details on the building software blocks developed, observing that the SDR-based system features a 10-fold improvement in the intrinsic sensitivity with respect to the original system tested, requiring 1/10 of the optical signal to attain the same performance. Indoors, the performances of the SDR-based full system were analyzed in the presence of different background light noise levels. By tuning the amount of background noise, we observe an improvement in the noise margin for error-free (PER $\leq 10^{-5})$ communications of 27 dB, attaining 40 dB in large-noise conditions. As our system is modular and could be replicated on existing VLC implementations, this makes virtually any VLC system much less prone to detrimental background light effects and variable signal levels, which are one of the key issues in indoor VLC applications.

In an outdoor scenario, under direct solar illuminance up to 12,000 lux, the SDR-based unit boosts the attainable error-free communication distance by a factor of 1.6 as compared to the original case, and successful communication up to 10 m is observed. As typical outdoor applications require VLC larger link lengths as compared to typical indoor cases, other factors, such as non-uniform surfaces, wind speed, and other environmental factors increase the difficulty in carrying tests outside. Our results are obtained by employing a commercial low-power V5W automotive LED lamp, and provide a clear validation of our SDR-based VLC system for realistic ITS applications, being able to achieve good PER performance under low SNR operating conditions, even in the presence of strong solar and artificial radiation. In conclusion, the use of DSP techniques significantly improves the performance of VLC receivers, improving resilience both to electronic noise and to that generated by background lights, and it is essential in the presence of broad-spectrum light sources, such as white LEDs, for which it is not possible to exploit an optical filtering solution. Our ongoing work focuses on the implementation of higher-order modulation schemes and filtering out multi-path effects, especially in short-range OWC using only a SDR.

## Figures and Tables

**Table 1:** Parameters used for the model and fit in Figure 3.

	*Full System* α=0.714		*Bridge System* α=1.25		*Arduino System* α=1.73	
	Model	Fit	Model	Fit	Model	Fit
Threshold $x_{c}$ (mV)	0	0 (fixed)	3.6	3.87	2.6	3.46
RMS noise *w* (mV)	0.14	0.15	1.0	1.63	1.0	2.38

## Data Availability

Not applicable.
